# Poly[[μ-aqua-tetraaquabis(μ-2-hydroxy-4-oxocyclobut-1-ene-1,3-diolato)strontium] hemihydrate]

**DOI:** 10.1107/S1600536811028704

**Published:** 2011-07-23

**Authors:** Amira Bouhali, Chahrazed Trifa, Sofiane Bouacida, Chaouki Boudaren, Thierry Bataille

**Affiliations:** a Unité de Recherche de Chimie de l’Environnement et Moléculaire Structurale, CHEMS, Université Mentouri–Constantine, 25000 Algeria; b Sciences Chimiques de Rennes, UMR 6226 CNRS - Université de Rennes 1, Avenue du Général Leclerc, 35042 Rennes cedex, France

## Abstract

In the title coordination polymer, {[Sr(C_4_HO_4_)_2_(H_2_O)_5_]·0.5H_2_O}_*n*_, the Sr^2+^ ion is coordinated by three monodentate hydrogensquarate (hsq) anions and six aqua ligands in a distorted SrO_9_ monocapped square-anti­prismatic geometry. The hsq anions and water mol­ecules bridge the metal ions into infinite sheets lying parallel to (100). The O atom of the uncoordinated water mol­ecule lies on a crystallographic twofold axis. The packing is stabilized by numerous O—H⋯O hydrogen bonds.

## Related literature

For the isostructural mixed-metal Ba/Sr analogue of the title compound and background references, see: Trifa *et al.* (2011 ▶). 
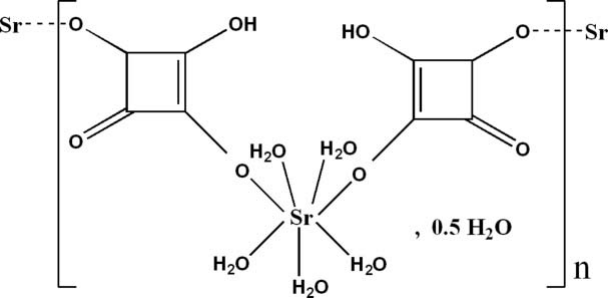


## Experimental

### 

#### Crystal data

[Sr(C_4_HO_4_)_2_(H_2_O)_5_]·0.5H_2_O
*M* *_r_* = 412.81
Monoclinic, 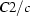

*a* = 24.885 (3) Å
*b* = 8.8026 (9) Å
*c* = 13.8918 (17) Å
β = 119.609 (4)°
*V* = 2645.7 (5) Å^3^
*Z* = 8
Mo *K*α radiation
μ = 4.15 mm^−1^
*T* = 150 K
0.57 × 0.27 × 0.10 mm

#### Data collection

Bruker APEXII diffractometer
Absorption correction: multi-scan *SADABS* (Bruker, 2006 ▶) *T*_min_ = 0.365, *T*_max_ = 0.660
9165 measured reflections
3006 independent reflections
2243 reflections with *I* > 2σ(*I*)
*R*_int_ = 0.037

#### Refinement

*R*[*F* ^2^ > 2σ(*F* ^2^)] = 0.028
*wR*(*F* ^2^) = 0.068
*S* = 1.03
3006 reflections
239 parameters
2 restraints
H atoms treated by a mixture of independent and constrained refinement
Δρ_max_ = 0.42 e Å^−3^
Δρ_min_ = −0.51 e Å^−3^

### 

Data collection: *APEX2* (Bruker, 2006 ▶); cell refinement: *SAINT* (Bruker, 2006 ▶); data reduction: *SAINT*; program(s) used to solve structure: *SIR2002* (Burla *et al.*, 2003 ▶); program(s) used to refine structure: *SHELXL97* (Sheldrick, 2008 ▶); molecular graphics: *ORTEP-3* (Farrugia, 1997 ▶) and *DIAMOND* (Brandenburg & Berndt, 2001 ▶); software used to prepare material for publication: *WinGX* publication routines (Farrugia, 1999 ▶).

## Supplementary Material

Crystal structure: contains datablock(s) global, I. DOI: 10.1107/S1600536811028704/hb5939sup1.cif

Structure factors: contains datablock(s) I. DOI: 10.1107/S1600536811028704/hb5939Isup2.hkl

Additional supplementary materials:  crystallographic information; 3D view; checkCIF report

## Figures and Tables

**Table 1:** Selected bond lengths (Å)

Sr1—O1	2.691 (2)
Sr1—O2	2.642 (2)
Sr1—O3	2.690 (2)
Sr1—O4	2.641 (3)
Sr1—O5	2.572 (2)
Sr1—O6	2.6179 (18)
Sr1—O12	2.6646 (16)
Sr1—O14^i^	2.5906 (16)
Sr1—O3^ii^	2.7154 (19)

Symmetry codes: (i) 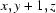; (ii) 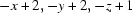.

**Table 2:** Hydrogen-bond geometry (Å, °)

*D*—H⋯*A*	*D*—H	H⋯*A*	*D*⋯*A*	*D*—H⋯*A*
O1—H1*A*⋯O9^iii^	0.76 (4)	2.27 (4)	2.983 (3)	158 (4)
O1—H1*B*⋯O7^iv^	0.79 (4)	1.99 (4)	2.715 (2)	153 (4)
O1*W*—H1*W*⋯O13^ii^	0.76 (2)	2.39 (2)	2.801 (2)	115 (2)
O1*W*—H1*W*⋯O8^v^	0.76 (2)	2.51 (3)	3.118 (2)	138 (3)
O2—H2*A*⋯O14^vi^	0.92 (4)	1.91 (4)	2.794 (3)	161 (4)
O2—H2*B*⋯O1*W*^vii^	0.70 (4)	2.52 (4)	3.165 (3)	154 (4)
O3—H3*A*⋯O13^ii^	0.93 (4)	1.79 (4)	2.712 (3)	174 (3)
O3—H3*B*⋯O4^viii^	0.76 (4)	2.59 (4)	3.172 (3)	136 (3)
O4—H4*A*⋯O7^ix^	0.78 (4)	2.01 (4)	2.785 (3)	177 (4)
O4—H4*B*⋯O1*W*	0.87 (4)	2.03 (4)	2.871 (3)	163 (3)
O5—H5*A*⋯O1^x^	0.72 (4)	2.09 (4)	2.787 (3)	166 (4)
O5—H5*B*⋯O8^xi^	0.90 (4)	1.82 (4)	2.716 (3)	175 (4)
O9—H9⋯O12	0.82	1.74	2.548 (3)	169
O11—H11⋯O6^vii^	0.82	1.77	2.580 (2)	172

Symmetry codes: (ii) 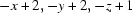; (iii) 
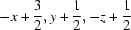
; (iv) 
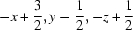
; (v) 
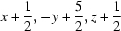
; (vi) 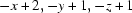; (vii) 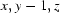; (viii) 
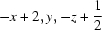
; (ix) 
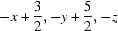
; (x) 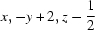; (xi) 
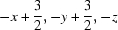
.

**Fig. 1.**
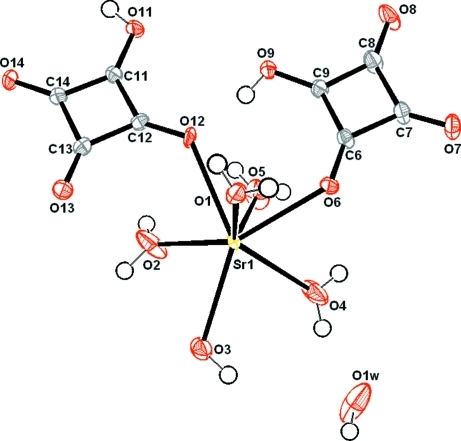
Drawing of asymetric unit of (I), with displacement ellipsoids drawn at the 50% probability level.

**Fig. 2.**
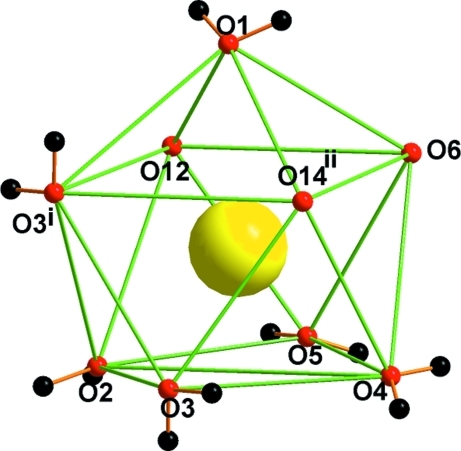
The monocapped square antiprism geometry of the Sr atom [Symmetry code: [(i):2 - x, 2 - y, 1 - z, (ii): x, 1 + y, z.].

**Fig. 3.**
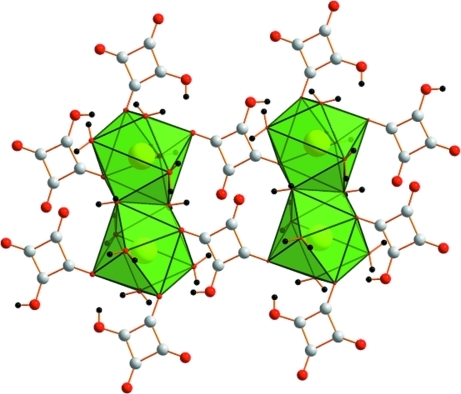
Coordination between squarates ligands and polyhedron unit

**Fig. 4.**
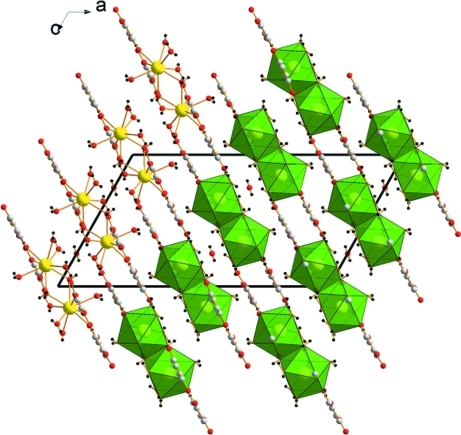
The crystal structure of the title compound viewed along the b axis, showing the layers.

## supplementary crystallographic information

### Comment

As part of our ongoing sturctural studies of alkaline-earth squarate coordination networks (Trifa *et al.*, 2011), we now descibe the synthesis and structure of the title compound, (I).

The asymmetric unit of (I) contains one strontium cation, two hydrogensquarate anions, five aqua ligands and a water molecule (Fig. 1): the O-atom of the latter lies on a crystallographic 2-fold axis.

The strontium atom is coordinated by the O atoms from three hydrogensquarate anions and six water molecules. The ninefold coordination polyhedral of Sr can be described as a monocapped square antiprism SrO_3_(H_2_O)_6_ (Fig. 2).

The hydrogensquarate (HSQ) anions bridge two strontium atoms to form a [Sr2 (H2O)12(HSQ)4] dimer (Fig. 3), thus leading a ribbon of formula [Sr (H2O)6(HC4O4)2] running along [101]. The SrO_9_ polyhedra are linked together by sharing two water molecules in the direction of the *b* axis to build up layers, in between which are located the free water molecules (Fig. 4). The three-dimensionality is ensured by a network of O—H···O hydrogen bonds (Table 2).

### Experimental

Board colourless single crystals of the title compound were obtained by a hydrothermal reaction of an equimolar ratio (1 mmol) of strontium chloride, squaric acid and and 6 ml of water in a 23 ml Teflon-lined acid digestion bomb (Parr) heated for 3 days at 393 K under autogeneous pressure and then cooled down to room temperature. The product obtained were collected by filtration, thoroughly washed with distilled water and ethanol, and finally dried at room temperature.

### Refinement

All non-H atoms were refined with anisotropic atomic displacement parameters. All H atoms were localized on Fourier maps and refined isotropically. Except for H atoms for hydroxy groups of hydrogenosquarate (H9 and H11) were introduced in calculated positions and treated as riding on their parent O atom.(with O—H = 0.82Å and *U*_iso_(H) =1.5*U*_eq_(O)), One distance (O—H)of free water molecule are refined with soft constraint, the O—H distance is restrained to 0.85 Å. (O1W—H1W).

### Crystal data

**Table:**

[Sr(C_4_HO_4_)_2_(H_2_O)_5_]·0.5H_2_O	*F*(000) = 1656
*M**_r_* = 412.81	*D*_x_ = 2.073 Mg m^−^^3^
Monoclinic, *C*2/*c*	Mo *K*α radiation, λ = 0.71073 Å
Hall symbol: -C 2yc	Cell parameters from 3634 reflections
*a* = 24.885 (3) Å	θ = 2.5–27.5°
*b* = 8.8026 (9) Å	µ = 4.15 mm^−^^1^
*c* = 13.8918 (17) Å	*T* = 150 K
β = 119.609 (4)°	Slab, colourless
*V* = 2645.7 (5) Å^3^	0.57 × 0.27 × 0.10 mm
*Z* = 8	

### Data collection

**Table:**

Bruker APEXII diffractometer	2243 reflections with *I* > 2σ(*I*)
graphite	*R*_int_ = 0.037
CCD rotation images, thin slices scans	θ_max_ = 27.5°, θ_min_ = 2.7°
Absorption correction: multi-scan *SADABS* (Bruker, 2006)	*h* = −32→32
*T*_min_ = 0.365, *T*_max_ = 0.660	*k* = −8→11
9165 measured reflections	*l* = −17→18
3006 independent reflections	

### Refinement

**Table:**

Refinement on *F*^2^	Primary atom site location: structure-invariant direct methods
Least-squares matrix: full	Secondary atom site location: difference Fourier map
*R*[*F*^2^ > 2σ(*F*^2^)] = 0.028	Hydrogen site location: inferred from neighbouring sites
*wR*(*F*^2^) = 0.068	H atoms treated by a mixture of independent and constrained refinement
*S* = 1.03	*w* = 1/[σ^2^(*F*_o_^2^) + (0.0273*P*)^2^ + 1.5645*P*] where *P* = (*F*_o_^2^ + 2*F*_c_^2^)/3
3006 reflections	(Δ/σ)_max_ = 0.001
239 parameters	Δρ_max_ = 0.42 e Å^−^^3^
2 restraints	Δρ_min_ = −0.51 e Å^−^^3^

### Special details

**Table:**

Geometry. Bond distances, angles *etc*. have been calculated using the rounded fractional coordinates. All su's are estimated from the variances of the (full) variance-covariance matrix. The cell e.s.d.'s are taken into account in the estimation of distances, angles and torsion angles
Refinement. Refinement of *F*^2^ against ALL reflections. The weighted *R*-factor *wR* and goodness of fit *S* are based on *F*^2^, conventional *R*-factors *R* are based on *F*, with *F* set to zero for negative *F*^2^. The threshold expression of *F*^2^ > σ(*F*^2^) is used only for calculating *R*-factors(gt) *etc*. and is not relevant to the choice of reflections for refinement. *R*-factors based on *F*^2^ are statistically about twice as large as those based on *F*, and *R*- factors based on ALL data will be even larger.

### Fractional atomic coordinates and isotropic or equivalent isotropic displacement parameters (Å^2^)

**Table:**

	*x*	*y*	*z*	*U*_iso_*/*U*_eq_	
Sr1	0.911042 (8)	0.99774 (2)	0.338611 (16)	0.00988 (8)	
O1	0.83711 (7)	1.00426 (19)	0.42892 (15)	0.0142 (3)	
O2	0.98788 (9)	0.7809 (2)	0.35213 (18)	0.0294 (5)	
O3	1.02876 (8)	1.0958 (2)	0.44716 (15)	0.0192 (4)	
O4	0.92432 (9)	1.2071 (3)	0.21578 (19)	0.0426 (7)	
O5	0.88485 (10)	0.8965 (3)	0.14660 (16)	0.0272 (5)	
O6	0.80135 (7)	1.10726 (17)	0.20264 (13)	0.0145 (4)	
O7	0.68196 (8)	1.24431 (17)	−0.01295 (14)	0.0167 (4)	
O8	0.63364 (7)	0.90554 (19)	−0.10260 (15)	0.0236 (4)	
O9	0.74969 (7)	0.76209 (17)	0.11403 (15)	0.0170 (4)	
H9	0.7835	0.763	0.1706	0.025*	
O11	0.80095 (7)	0.40021 (18)	0.20800 (14)	0.0166 (4)	
H11	0.8005	0.3073	0.2116	0.025*	
O12	0.84872 (7)	0.73793 (16)	0.29979 (14)	0.0144 (4)	
O13	0.95937 (8)	0.5984 (2)	0.52759 (16)	0.0318 (5)	
O14	0.91536 (7)	0.25641 (17)	0.43112 (15)	0.0207 (4)	
C6	0.75625 (10)	1.0501 (3)	0.1175 (2)	0.0114 (5)	
C7	0.70216 (10)	1.1153 (3)	0.0187 (2)	0.0130 (5)	
C8	0.67972 (11)	0.9580 (3)	−0.0218 (2)	0.0159 (5)	
C9	0.73365 (10)	0.9012 (3)	0.0776 (2)	0.0126 (5)	
C11	0.84871 (10)	0.4537 (3)	0.2975 (2)	0.0135 (5)	
C12	0.86769 (10)	0.6055 (3)	0.3350 (2)	0.0130 (5)	
C13	0.91914 (11)	0.5446 (3)	0.4401 (2)	0.0206 (6)	
C14	0.89846 (10)	0.3877 (3)	0.3960 (2)	0.0165 (5)	
O1W	1	1.4700 (3)	0.25	0.0446 (9)	
H1B	0.8227 (16)	0.926 (4)	0.432 (3)	0.05*	
H1A	0.8113 (17)	1.062 (4)	0.401 (3)	0.05*	
H5B	0.8812 (15)	0.796 (4)	0.134 (3)	0.05*	
H2A	1.0251 (15)	0.776 (4)	0.417 (3)	0.05*	
H5A	0.8711 (17)	0.934 (5)	0.094 (3)	0.05*	
H3B	1.0439 (16)	1.072 (4)	0.414 (3)	0.05*	
H3A	1.0298 (15)	1.201 (4)	0.453 (3)	0.05*	
H2B	0.9798 (16)	0.719 (4)	0.316 (3)	0.05*	
H4A	0.8953 (17)	1.219 (4)	0.158 (3)	0.05*	
H4B	0.9498 (16)	1.284 (4)	0.241 (3)	0.05*	
H1W	1.0225 (13)	1.513 (3)	0.3020 (13)	0.05*	

### Atomic displacement parameters (Å^2^)

**Table:**

	*U*^11^	*U*^22^	*U*^33^	*U*^12^	*U*^13^	*U*^23^
Sr1	0.00824 (11)	0.00952 (13)	0.00876 (12)	0.00008 (8)	0.00180 (8)	0.00180 (9)
O1	0.0145 (8)	0.0109 (9)	0.0172 (9)	0.0003 (7)	0.0078 (7)	0.0015 (8)
O2	0.0159 (9)	0.0414 (13)	0.0193 (11)	0.0073 (9)	−0.0001 (8)	−0.0139 (9)
O3	0.0145 (8)	0.0236 (10)	0.0162 (10)	−0.0018 (8)	0.0050 (7)	0.0060 (8)
O4	0.0184 (10)	0.0565 (15)	0.0300 (13)	−0.0138 (10)	−0.0057 (9)	0.0294 (12)
O5	0.0345 (11)	0.0293 (11)	0.0111 (10)	0.0049 (9)	0.0062 (9)	−0.0023 (9)
O6	0.0125 (7)	0.0102 (8)	0.0112 (9)	−0.0020 (6)	−0.0015 (7)	−0.0015 (7)
O7	0.0195 (9)	0.0117 (9)	0.0123 (9)	0.0056 (7)	0.0028 (7)	0.0001 (7)
O8	0.0161 (8)	0.0192 (9)	0.0174 (10)	0.0049 (7)	−0.0056 (8)	−0.0093 (8)
O9	0.0125 (8)	0.0095 (8)	0.0179 (10)	−0.0005 (6)	−0.0008 (7)	0.0016 (7)
O11	0.0128 (8)	0.0093 (8)	0.0157 (9)	−0.0018 (7)	−0.0020 (7)	−0.0020 (7)
O12	0.0158 (8)	0.0052 (8)	0.0142 (9)	0.0025 (6)	0.0014 (7)	0.0023 (7)
O13	0.0286 (10)	0.0163 (9)	0.0199 (11)	−0.0104 (8)	−0.0113 (8)	0.0048 (8)
O14	0.0169 (9)	0.0069 (8)	0.0245 (11)	0.0008 (6)	−0.0003 (8)	0.0040 (7)
C6	0.0097 (10)	0.0124 (11)	0.0106 (12)	0.0029 (8)	0.0038 (9)	−0.0007 (9)
C7	0.0131 (11)	0.0132 (12)	0.0098 (12)	0.0042 (9)	0.0035 (10)	−0.0006 (10)
C8	0.0156 (11)	0.0136 (12)	0.0135 (13)	0.0038 (9)	0.0033 (10)	−0.0036 (10)
C9	0.0106 (10)	0.0093 (11)	0.0146 (12)	0.0011 (9)	0.0038 (10)	−0.0018 (10)
C11	0.0110 (10)	0.0085 (10)	0.0152 (13)	0.0003 (8)	0.0021 (10)	0.0000 (10)
C12	0.0132 (11)	0.0071 (11)	0.0142 (13)	−0.0013 (9)	0.0034 (10)	−0.0020 (10)
C13	0.0178 (12)	0.0104 (11)	0.0209 (15)	−0.0033 (9)	−0.0001 (11)	0.0031 (10)
C14	0.0121 (11)	0.0102 (11)	0.0185 (14)	−0.0014 (9)	0.0008 (10)	0.0016 (11)
O1W	0.095 (3)	0.0180 (16)	0.0227 (17)	0	0.031 (2)	0

### Geometric parameters (Å, °)

**Table:**

Sr1—O1	2.691 (2)	O2—H2B	0.70 (4)
Sr1—O2	2.642 (2)	O3—H3B	0.76 (4)
Sr1—O3	2.690 (2)	O3—H3A	0.93 (4)
Sr1—O4	2.641 (3)	O4—H4B	0.87 (4)
Sr1—O5	2.572 (2)	O4—H4A	0.78 (4)
Sr1—O6	2.6179 (18)	O5—H5A	0.72 (4)
Sr1—O12	2.6646 (16)	O5—H5B	0.90 (4)
Sr1—O14^i^	2.5906 (16)	O9—H9	0.8200
Sr1—O3^ii^	2.7154 (19)	O11—H11	0.8200
O6—C6	1.265 (3)	O1W—H1W	0.76 (2)
O7—C7	1.232 (3)	O1W—H1W^iii^	0.76 (2)
O8—C8	1.231 (3)	C6—C7	1.483 (4)
O9—C9	1.310 (3)	C6—C9	1.426 (4)
O11—C11	1.311 (3)	C7—C8	1.495 (4)
O12—C12	1.262 (3)	C8—C9	1.458 (4)
O13—C13	1.225 (3)	C11—C14	1.438 (4)
O14—C14	1.244 (3)	C11—C12	1.427 (4)
O1—H1A	0.76 (4)	C12—C13	1.487 (4)
O1—H1B	0.79 (4)	C13—C14	1.495 (4)
O2—H2A	0.92 (4)		
			
O1—Sr1—O2	128.30 (6)	Sr1—O1—H1A	112 (3)
O1—Sr1—O3	122.53 (6)	H2A—O2—H2B	117 (4)
O1—Sr1—O4	128.24 (7)	Sr1—O2—H2A	117 (2)
O1—Sr1—O5	127.55 (7)	Sr1—O2—H2B	125 (3)
O1—Sr1—O6	67.62 (6)	H3A—O3—H3B	109 (4)
O1—Sr1—O12	69.46 (5)	Sr1—O3—H3B	108 (3)
O1—Sr1—O14^i^	67.69 (6)	Sr1—O3—H3A	110 (3)
O1—Sr1—O3^ii^	68.26 (6)	Sr1^ii^—O3—H3A	103 (2)
O2—Sr1—O3	69.05 (6)	Sr1^ii^—O3—H3B	114 (3)
O2—Sr1—O4	103.47 (8)	Sr1—O4—H4A	115 (3)
O2—Sr1—O5	68.10 (7)	Sr1—O4—H4B	125 (2)
O2—Sr1—O6	141.02 (6)	H4A—O4—H4B	115 (4)
O2—Sr1—O12	73.90 (6)	Sr1—O5—H5A	130 (3)
O2—Sr1—O14^i^	138.47 (6)	Sr1—O5—H5B	120 (2)
O2—Sr1—O3^ii^	73.44 (6)	H5A—O5—H5B	108 (4)
O3—Sr1—O4	71.88 (7)	C9—O9—H9	109.00
O3—Sr1—O5	109.90 (7)	C11—O11—H11	109.00
O3—Sr1—O6	138.14 (5)	H1W—O1W—H1W^iii^	120 (3)
O3—Sr1—O12	138.82 (5)	O6—C6—C7	133.8 (2)
O3—Sr1—O14^i^	70.92 (6)	O6—C6—C9	136.6 (2)
O3—Sr1—O3^ii^	67.95 (6)	C7—C6—C9	89.6 (2)
O4—Sr1—O5	67.68 (8)	O7—C7—C6	135.3 (2)
O4—Sr1—O6	72.28 (7)	C6—C7—C8	89.4 (2)
O4—Sr1—O12	135.59 (6)	O7—C7—C8	135.1 (2)
O4—Sr1—O14^i^	73.70 (7)	O8—C8—C7	134.2 (2)
O3^ii^—Sr1—O4	137.93 (7)	O8—C8—C9	137.9 (2)
O5—Sr1—O6	74.91 (7)	C7—C8—C9	87.9 (2)
O5—Sr1—O12	70.66 (7)	C6—C9—C8	93.1 (2)
O5—Sr1—O14^i^	138.26 (7)	O9—C9—C6	136.3 (2)
O3^ii^—Sr1—O5	138.71 (7)	O9—C9—C8	130.4 (2)
O6—Sr1—O12	82.82 (5)	C12—C11—C14	93.3 (2)
O6—Sr1—O14^i^	78.88 (5)	O11—C11—C12	131.6 (2)
O3^ii^—Sr1—O6	135.76 (6)	O11—C11—C14	135.1 (2)
O12—Sr1—O14^i^	137.07 (6)	O12—C12—C13	133.6 (2)
O3^ii^—Sr1—O12	84.97 (5)	O12—C12—C11	136.9 (2)
O3^ii^—Sr1—O14^i^	81.82 (5)	C11—C12—C13	89.4 (2)
Sr1—O3—Sr1^ii^	112.05 (7)	O13—C13—C12	136.0 (2)
Sr1—O6—C6	130.94 (16)	O13—C13—C14	135.3 (2)
Sr1—O12—C12	130.20 (17)	C12—C13—C14	88.6 (2)
Sr1^iv^—O14—C14	134.33 (16)	O14—C14—C13	135.7 (2)
Sr1—O1—H1B	116 (3)	C11—C14—C13	88.7 (2)
H1A—O1—H1B	109 (4)	O14—C14—C11	135.5 (2)
			
O1—Sr1—O3—Sr1^ii^	42.91 (9)	Sr1—O6—C6—C7	−157.2 (2)
O2—Sr1—O3—Sr1^ii^	−79.97 (8)	Sr1—O6—C6—C9	26.7 (5)
O4—Sr1—O3—Sr1^ii^	167.22 (9)	Sr1—O12—C12—C11	−152.0 (3)
O5—Sr1—O3—Sr1^ii^	−135.69 (8)	Sr1—O12—C12—C13	32.5 (4)
O6—Sr1—O3—Sr1^ii^	134.93 (7)	Sr1^iv^—O14—C14—C11	32.9 (5)
O12—Sr1—O3—Sr1^ii^	−52.65 (11)	Sr1^iv^—O14—C14—C13	−150.9 (3)
O14^i^—Sr1—O3—Sr1^ii^	88.72 (7)	O6—C6—C7—O7	−2.1 (6)
O3^ii^—Sr1—O3—Sr1^ii^	0.00 (6)	O6—C6—C7—C8	−177.7 (3)
O1—Sr1—O6—C6	−106.5 (2)	C9—C6—C7—O7	175.2 (3)
O2—Sr1—O6—C6	17.3 (3)	C9—C6—C7—C8	−0.4 (2)
O3—Sr1—O6—C6	139.1 (2)	O6—C6—C9—O9	1.8 (6)
O4—Sr1—O6—C6	106.9 (2)	O6—C6—C9—C8	177.6 (3)
O5—Sr1—O6—C6	36.0 (2)	C7—C6—C9—O9	−175.3 (3)
O12—Sr1—O6—C6	−35.8 (2)	C7—C6—C9—C8	0.5 (2)
O14^i^—Sr1—O6—C6	−176.8 (2)	O7—C7—C8—O8	2.0 (6)
O3^ii^—Sr1—O6—C6	−111.0 (2)	O7—C7—C8—C9	−175.2 (3)
O1—Sr1—O12—C12	−112.7 (2)	C6—C7—C8—O8	177.6 (3)
O2—Sr1—O12—C12	30.1 (2)	C6—C7—C8—C9	0.4 (2)
O3—Sr1—O12—C12	3.6 (2)	O8—C8—C9—O9	−1.3 (6)
O4—Sr1—O12—C12	123.1 (2)	O8—C8—C9—C6	−177.4 (4)
O5—Sr1—O12—C12	102.0 (2)	C7—C8—C9—O9	175.7 (3)
O6—Sr1—O12—C12	178.5 (2)	C7—C8—C9—C6	−0.5 (2)
O14^i^—Sr1—O12—C12	−116.4 (2)	O11—C11—C12—O12	−1.5 (6)
O3^ii^—Sr1—O12—C12	−44.1 (2)	O11—C11—C12—C13	175.3 (3)
O1—Sr1—O14^i^—C14^i^	−112.4 (3)	C14—C11—C12—O12	−178.2 (3)
O2—Sr1—O14^i^—C14^i^	124.5 (2)	C14—C11—C12—C13	−1.4 (2)
O3—Sr1—O14^i^—C14^i^	108.4 (3)	O11—C11—C14—O14	2.3 (6)
O4—Sr1—O14^i^—C14^i^	32.4 (3)	O11—C11—C14—C13	−175.1 (3)
O5—Sr1—O14^i^—C14^i^	9.6 (3)	C12—C11—C14—O14	178.8 (3)
O6—Sr1—O14^i^—C14^i^	−42.2 (3)	C12—C11—C14—C13	1.4 (2)
O12—Sr1—O14^i^—C14^i^	−108.7 (2)	O12—C12—C13—O13	1.5 (6)
O1—Sr1—O3^ii^—Sr1^ii^	−141.83 (8)	O12—C12—C13—C14	178.3 (3)
O2—Sr1—O3^ii^—Sr1^ii^	73.62 (8)	C11—C12—C13—O13	−175.4 (4)
O3—Sr1—O3^ii^—Sr1^ii^	0.00 (6)	C11—C12—C13—C14	1.4 (2)
O4—Sr1—O3^ii^—Sr1^ii^	−18.28 (14)	O13—C13—C14—O14	−1.9 (6)
O5—Sr1—O3^ii^—Sr1^ii^	95.46 (11)	O13—C13—C14—C11	175.5 (4)
O6—Sr1—O3^ii^—Sr1^ii^	−137.38 (7)	C12—C13—C14—O14	−178.7 (3)
O12—Sr1—O3^ii^—Sr1^ii^	148.30 (8)	C12—C13—C14—C11	−1.4 (2)

Symmetry codes: (i) *x*, *y*+1, *z*; (ii) −*x*+2, −*y*+2, −*z*+1; (iii) −*x*+2, *y*, −*z*+1/2; (iv) *x*, *y*−1, *z*.

### Hydrogen-bond geometry (Å, °)

**Table:**

*D*—H···*A*	*D*—H	H···*A*	*D*···*A*	*D*—H···*A*
O1—H1A···O9^v^	0.76 (4)	2.27 (4)	2.983 (3)	158 (4)
O1—H1B···O7^vi^	0.79 (4)	1.99 (4)	2.715 (2)	153 (4)
O1W—H1W···O13^ii^	0.76 (2)	2.39 (2)	2.801 (2)	115 (2)
O1W—H1W···O8^vii^	0.76 (2)	2.51 (3)	3.118 (2)	138 (3)
O2—H2A···O14^viii^	0.92 (4)	1.91 (4)	2.794 (3)	161 (4)
O2—H2B···O1W^iv^	0.70 (4)	2.52 (4)	3.165 (3)	154 (4)
O3—H3A···O13^ii^	0.93 (4)	1.79 (4)	2.712 (3)	174 (3)
O3—H3B···O4^iii^	0.76 (4)	2.59 (4)	3.172 (3)	136 (3)
O4—H4A···O7^ix^	0.78 (4)	2.01 (4)	2.785 (3)	177 (4)
O4—H4B···O1W	0.87 (4)	2.03 (4)	2.871 (3)	163 (3)
O5—H5A···O1^x^	0.72 (4)	2.09 (4)	2.787 (3)	166 (4)
O5—H5B···O8^xi^	0.90 (4)	1.82 (4)	2.716 (3)	175 (4)
O9—H9···O12	0.82	1.74	2.548 (3)	169
O11—H11···O6^iv^	0.82	1.77	2.580 (2)	172

Symmetry codes: (v) −*x*+3/2, *y*+1/2, −*z*+1/2; (vi) −*x*+3/2, *y*−1/2, −*z*+1/2; (ii) −*x*+2, −*y*+2, −*z*+1; (vii) *x*+1/2, −*y*+5/2, *z*+1/2; (viii) −*x*+2, −*y*+1, −*z*+1; (iv) *x*, *y*−1, *z*; (iii) −*x*+2, *y*, −*z*+1/2; (ix) −*x*+3/2, −*y*+5/2, −*z*; (x) *x*, −*y*+2, *z*−1/2; (xi) −*x*+3/2, −*y*+3/2, −*z*.
